# The plant ARF GTPase family: current perspectives in membrane trafficking, cellular signaling, and new paradigms

**DOI:** 10.1093/pcp/pcag079

**Published:** 2026-06-22

**Authors:** Inga Mohr, Sibaji K Sanyal, Rumen Ivanov, Petra Bauer

**Affiliations:** Institute of Botany, Heinrich Heine University, Universitätsstr. 1, 40225 Düsseldorf, Germany; Institute of Botany, Heinrich Heine University, Universitätsstr. 1, 40225 Düsseldorf, Germany; Institute of Botany, Heinrich Heine University, Universitätsstr. 1, 40225 Düsseldorf, Germany; Institute of Botany, Heinrich Heine University, Universitätsstr. 1, 40225 Düsseldorf, Germany; Cluster of Excellence on Plant Science (CEPLAS), Heinrich-Heine-University, Universitätsstr. 1, 40225 Düsseldorf, Germany

**Keywords:** small GTPases, ADP-ribosylation factor, ARF-like, secretion-associated and Ras-superfamily related, guanine nucleotide exchange factors, GNOM, BIG, GTPase-activating proteins

## Abstract

Plant ADP-ribosylation factor (ARF) and ARF-like (ARL) proteins are subfamily members of the small GTPases superfamily. They are pivotal regulators of dynamic cellular processes, notably vesicle trafficking, through their ability to switch conformations between GTP- and GDP-bound states. Despite their high conservation across species, the diversity and functions of ARF/ARL proteins, along with their regulatory guanine nucleotide exchange factors (GEFs) and GTPase-activating proteins (GAPs), vary considerably among organisms, reflecting species-specific roles. In plants, as compared to mammals, the ARF subfamily and its cognate GEFs and the GAPs family remain understudied. Recent advances have begun to unveil their biochemical activities and physiological significance in plant development and stress responses. This review synthesizes current knowledge on the plant ARF subfamily, emphasizing their regulatory interactions and physiological contexts, and highlights avenues for future research into their roles in cell physiological signaling pathways critical for plant growth and adaptation.

## Introduction

Small guanine-nucleotide-binding GTPase proteins act as molecular switches in a broad range of regulatory signal transduction events. These small GTPases belong to the Ras superfamily (Bos 1988, Hall 1990, Kahn et al. 1992). Ras superfamily proteins exhibit consensus motifs for GTP-binding and GTPase activity and have a molecular mass around 20−29 kDa (Kahn et al. 1992, Ahmadi et al. 2017). The Ras family is grouped into different subfamilies based on criteria such as sequence similarity and characteristic sequence motifs at the protein termini. In mammals, the Ras superfamily comprises five major subfamilies: Ras sarcoma (RAS), Ras homologs (RHO), Ras-like proteins in brain (RAB), Ras-related nuclear proteins (RAN), and ADP-ribosylation factor (ARF) (Kahn et al. 1992, Ahmadi et al. 2017). The differences between ARFs and other RAS proteins, e.g. regarding intrinsic GTPase rates, have suggested that ARFs vary substantially from other RAS proteins (Turn et al. 2025). As plants lack canonical Ras subfamily members, the small GTPase superfamily is the more accepted terminology. The model plant species *Arabidopsis thaliana* (*Arabidopsis*) is the reference species for flowering plants and serves as a blueprint in the green lineage. *Arabidopsis* has four subfamilies within the small GTPase superfamily: Rho of plants (ROP), RAB, RAN, and ARF (Vernoud et al. 2003) (Fig. 1a). This classification is applicable to other plant species as well (Nielsen 2020). The cellular and physiological roles of plant GTPases have been discussed elsewhere (McElver et al. 2000, Hwang and Robinson 2009, Yorimitsu et al. 2014, Singh and Jurgens 2018, Nielsen 2020, Shi et al. 2023, Denninger 2024, Lu et al. 2024).

**Figure 1:**
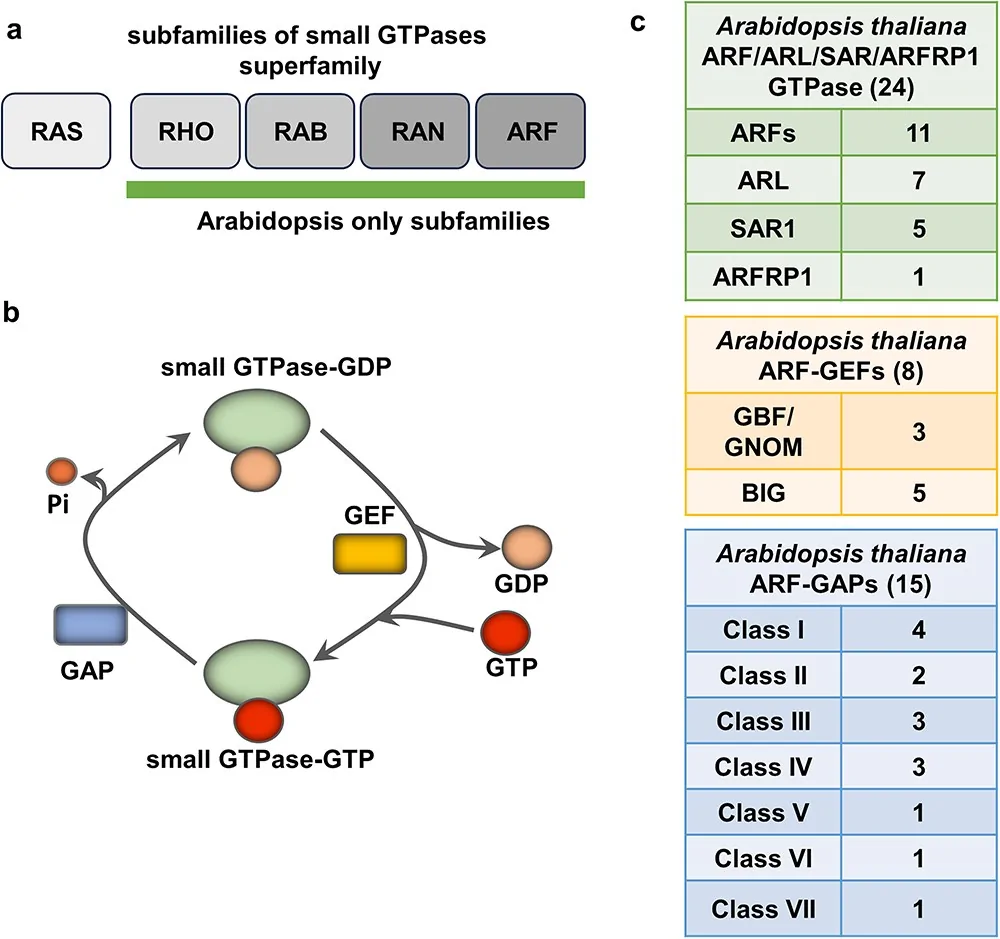
Small GTPases- classification of subfamilies, their nucleotide exchange cycle, and ARF GTPases in *A. thaliana*. (a) A total of five major subfamilies are present in humans, while in *Arabidopsis*, four are present (denoted by a green bar). The *Arabidopsis* Rho family is known as ROP. (b) GTPases exist in either an inactive GDP-bound or an active GTP-bound state. Oftentimes, due to low internal dissociation and hydrolysis rates, the enzymatic reactions are aided by special proteins. GEFs help to release the bound GDP to enable the exchange to GTP, whereas GAPs catalyze GTP hydrolysis. (c) Classification and class-specific member counts of ARF GTPases, GEFs, and GAPs in *A. thaliana*. Only ARF-GEFs and ARF-GAPs are indicated; SAR-GEFs and SAR-GAPs are not included. Table 1 includes the *Arabidopsis* locus IDs of all the genes mentioned in the figure.

Small GTPases function as a molecular switch by alternating between an inactive GDP-bound and an active GTP-bound state. This conformational switching is regulated in many cases by additional proteins due to the low intrinsic nucleotide exchange and hydrolysis activity of most of the small GTPases (Fig. 1b). Their activation as GTPase is facilitated by guanine nucleotide exchange factors (GEFs), which decrease the affinity of small GTPases for GDP and promote GTP exchange (Vernoud et al. 2003), while GTPase-activating proteins (GAPs) help in increasing the rate of hydrolysis of the bound GTP. Such behaviors can be studied by biochemical GTPase activity assays (Eberth and Ahmadian 2009, Jaiswal et al. 2012). Research from mammalian systems has indicated that some non-canonical proteins can also perform functions of GEFs (e.g. AMP-activated protein kinase) or GAPs (e.g. cell engulfment and motility domain proteins) (Nikolatou et al. 2023). There is no concrete evidence of such non-canonical GEF/GAP proteins in plants. Some GTPases may differ and be less dependent on GEFs or GAPs or be additionally controlled by guanine dissociation inhibitors (GDIs) or GDI-like proteins (Nielsen 2020).

Differences between the small GTPase subfamilies relate furthermore to isoprenyl decorations for membrane insertion. The Ras, RHO, and RAB proteins are prenylated at their C-termini hypervariable regions (Toma-Fukai and Shimizu 2019). RAB proteins end in CC/CXC/CCX/CCXX/CCXXX motifs that undergo geranylgeranyl modification (Ganotra et al. 2023). The ROPs show both CAAX motif and geranylgeranyl isoprenylation (Type I ROPs) and a C-terminus GC-CG box that can be S-acetylated (Type II ROPs) (Denninger 2024). The RANs have conserved acidic domains (DDDDD/E) at their C-terminus and do not undergo post-translational lipid modification (Nielsen 2020). For ARF proteins, the lipid modification site is a conserved Gly-2 residue at the N-terminus, which undergoes N-myristoylation (Kahn et al. 1992, Ahmadi et al. 2017).

In comparison to mammalian ARFs, very little is known about the plant ARF subfamily. The *Arabidopsis* ARF subfamily comprises 24 proteins, including 11 ARF proteins, 7 ARF-like (ARL) proteins, 5 secretion-associated and Ras-superfamily related (SAR) proteins, and additionally 1 ARF-related protein ARFRP1/ATARLB1. Furthermore, ARF subfamily proteins are supported by 8 ARF-GEFs and 15 ARF-GAPs in *Arabidopsis* (Fig. 1c). The SAR-GTPases are supported by three SAR-GEFs and seven SAR-GAPs. The ARF subfamily proteins are evolutionarily conserved (in terms of sequence similarity) (Vargova et al. 2025). However, there are differences between plant and animal endomembrane trafficking machinery functionalities [e.g. a mobile Golgi in plants*,* the *trans*-Golgi Network (TGN) as a central cargo sorting station, a (putative/debated) recycling endosome (RE), formation of cell plate for cytokinesis in plants, a plant specific T-Plate complex] (Gadeyne et al. 2014, Brandizzi 2018, Qi et al. 2024, Sinclair et al. 2024). Since the ARF subfamily plays a major role in endomembrane trafficking and there are the inherent differences between animals and plants, it is important to create a plant-specific ARF GTPase paradigm. Here, we provide an overview of the current understanding of *Arabidopsis* and other plant ARF GTPases and their regulators. For readers interested in the current state of plant endomembrane trafficking (endocytosis, recycling, and degradation), we refer to recently published reviews by Rodriguez-Furlan et al. (2019), Aniento et al. (2022), Zouhar et al. (2023), and Qi et al. (2024).

## Conserved Residues and Molecular Switch Mechanism of ARF Proteins

This detailed review chronicles ARF discovery (Turn et al. 2025). Mammalian models suggest that nucleotide binding is achieved by five conserved guanine-nucleotide-binding motifs or G-boxes (named G1 to G5), where each G-box has a consensus amino acid sequence. The G1 motif (also known as the Walker A motif/P loop) with GXXXXGKS/T binds the β- and γ-phosphate groups of GTP and catalyzes the phosphate transfer. The G2 (with a conserved Thr) is needed for Mg^2+^-binding. The G3 motif with DXXG is involved in Mg^2+^-binding via the Asp residue and γ-phosphate-binding via the Gly residue. The G4 with N/TKXD includes Lys and Asp that bind directly to nucleotides, while G5 with T/GC/SA is involved in guanine base recognition. The *Arabidopsis* ARF GTPase sequence comparison shows that these motifs are present (Fig. 2a). Two mutations in key residues in the G motifs are routinely employed to investigate the biochemical and physiological roles of ARFs. First, the substitution of Thr (at position 31 in Human and *Arabidopsis* ARF1) in G1 motif with Asn inhibits GDP−GTP exchange, making the ARF mutant a “dominant-negative” version of the protein (Hall 1990, Dascher and Balch 1994). The second mutation, Gln (at position 71 in Human and *Arabidopsis* ARF1) of G3 to Leu, results in a “dominant-active” version of the protein (a state where GTP hydrolysis is slowed down/aka Q70L), (study performed with *Bos taurus* ARF1) (Dascher and Balch 1994, Scheffzek et al. 1997) (Fig. 2a). The dominant-active mutation could lead to uncontrolled coat proteins [COAT PROTEIN (COP)] recruitment or disassembly. It may also cause the formation of unusually large vesicles and/or affect organelle structure. These can lead to disruptions in protein trafficking. The results of dominant-negative and dominant-active *Arabidopsis* ARF1 mutations are documented in Singh et al. (2018). Both mutations can lead to deleterious effects on cargo transport from the donor to the acceptor membrane. The spatial positions of the motifs can be seen in the example of *Homo sapiens* (Hs) HsARF6 (Fig. 2b and c) (Menetrey et al. 2000). The conformational change of the two switch regions (Fig. 2c), switch I (around G2) and switch II (containing G3), is important for GTP-binding. The movement allows the formation of two hydrogen bonds between the γ-phosphate and the Thr in switch I and the Gly in switch II. GTP hydrolysis leads to the relaxation of these two regions (Pai et al. 1989, Vetter and Wittinghofer 2001). The interswitch is a linker between the two switches and the N-terminal amphipathic helix (Fig. 2c). Superimposition of AlphaFold-predicted structures of AtARF1 on HsARF6 suggests that AtARF1 can match the conformation of HsARF6 (Fig. 2b and c).

**Figure 2:**
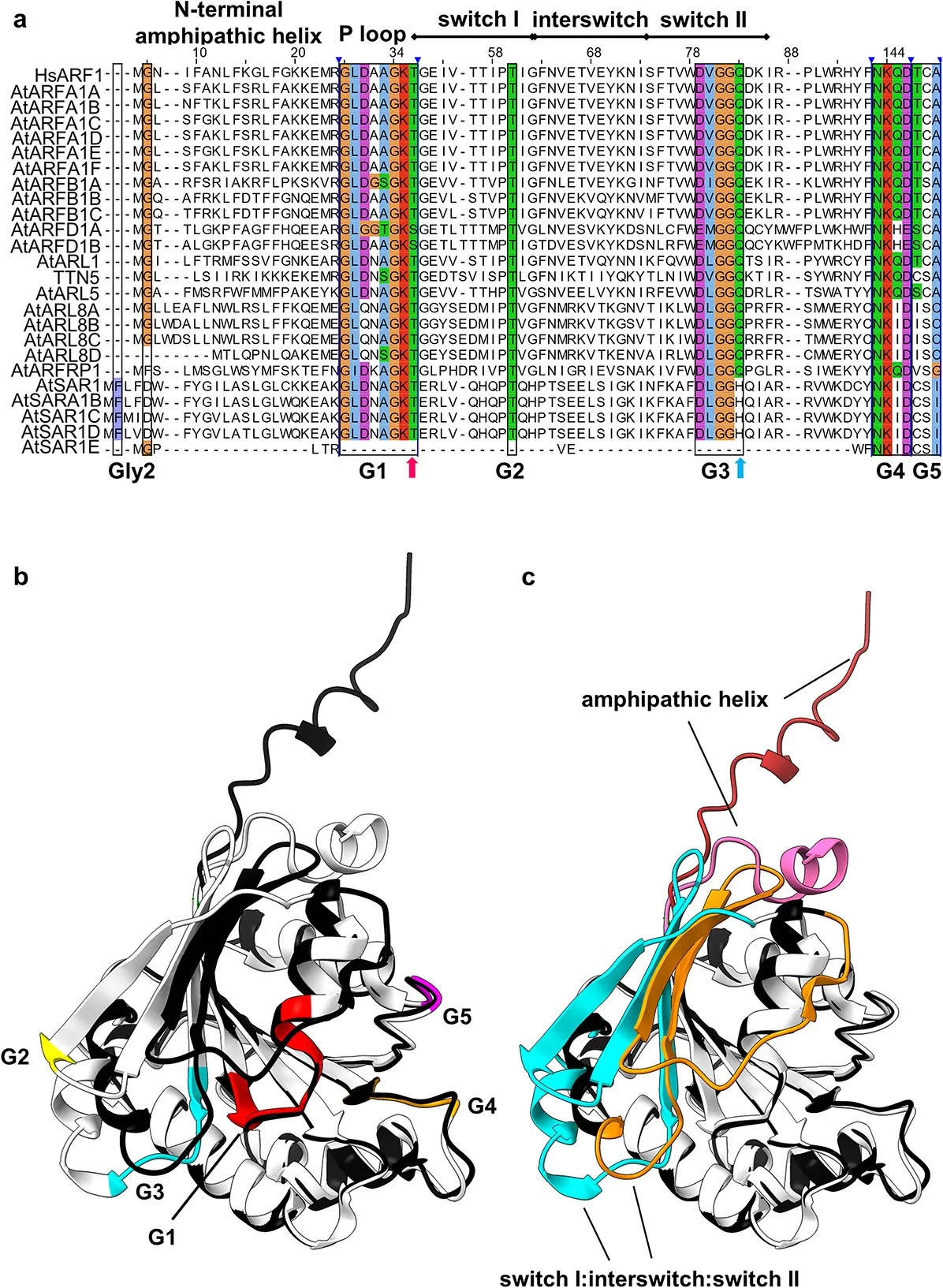
The *Arabidopsis* ARF family—motifs, domains, and 3D structure. (a) Sequence alignment of *Arabidopsis* ARF, ARL, and SAR proteins with HsARF1. Most possess a conserved Gly at position 2, as a potential myristoylation site. Next are the conserved G-binding motifs G1 (GXXXXGKS/T), G2 [Thr (T)], G3 (DXXG), G4 (N/TKXD), and G5 (T/GC/SA). Switch I and switch II are important regions for the conformational change that allows GTP binding. Interswitch toggle between switch I and switch II allows communication between the helix and the nucleotide-binding site. Mutations at specific conserved residues lead to the GTPase inactivity or activity. Inactivity: Thr to Asn (T to N, indicated by a red arrow in the G1 motif), activity: Gln to Lys (Q to L, indicated by a blue arrow in the G3 motif). Only important motifs are represented in the alignment. AtSAR1E may be a pseudogene. (b, c) Superimposed images of the crystal structure of HsARF6 (1E0S, Menetrey et al. 2000, in dull white) and the AlphaFold predicted structure of AtARF1 (P36397, in black, with an average pLDDT of 82.62%), edited with ChimeraX (1.11.1, Goddard et al. 2017). Root Mean Square Deviation 0.7 angstroms. Note that for P36397, N-terminal and the switch I: Interswitch: switch II regions are predicted with low confidence, and may be a possible reason of imperfect superimposition at these regions. (b) The G-binding motifs G1, G2, G3, G4, and G5 are indicated. (c) The entire switch region, comprising switch I, the interswitch, and switch II, (in cyan for HsARF6 and in orange for AtARF1), and the amphipathic helix (in magenta for HsARF6 and in fire brick for AtARF1), are indicated.

ARF proteins (inferred from HsARF1) differ in their conformation from other RAS members in their inactive state (Goldberg 1998). In the GTP-bound form, HsARF1 resembles the conformation of RAS-GTP, whereas the GDP-bound form differs due to its N-terminal amphipathic helix, which is myristoylated and mediates membrane anchoring (Goldberg 1998). In the GDP-bound conformation of HsARF1, the N-terminal helix lies in a shallow groove resulting from an inward movement of the interswitch. The GTP-bound conformation is achieved by reorganization of key amino acids, resulting in stabilization of switch II by the formation of a network of hydrogen bonds and the N-terminal helix is pushed from its binding pocket by the interswitch (Pasqualato et al. 2002). When GTP is bound, the γ-phosphate of GTP is coordinated via hydrogen bonds with Thr and Gly of switch I and II, respectively, with Thr contributing in Mg^2+^-binding by its side chain (Vetter and Wittinghofer 2001, Wittinghofer and Vetter 2011).

This N-terminal amphipathic helix mediates membrane association and appears to be evolutionarily conserved, as observed in ARF-related proteins from the Promethearchaeati (previously Asgard) archaea superphylum (Zaremba-Niedzwiedzka et al. 2017, Vargova et al. 2025). Recently, the membrane association of HsARF1 through its N-terminal myristoylated amphipathic helix and the structural rearrangement has been described using nuclear magnetic resonance, neutron reflectometry, and molecular dynamics simulations (Zhang et al. 2023). But, it is also important to note that some ARF family members utilize alternative mechanisms for membrane targeting (Vargova et al. 2021).

## ARF and SAR Proteins in Plants: Recruiting COPI and COPII

ARF GTPases can regulate COPI and COPII recruitment for vesicle formation during various membrane trafficking events. ARL subfamily members add more variations to this function, e.g. being involved in microtubule (MT) biogenesis and protein recruitment. Some mammalian ARL functions (like helping in motor movement) still lack corresponding evidence in plants (Sztul et al. 2019). Readers are directed to these reviews, which summarize the cellular localization and functions of the mammalian ARF subfamily (Sztul et al. 2019, Adarska et al. 2021). Note that in this paragraph section, “ARF” refers to the 11 ARF members of the ARF subfamily (Fig. 1c, Table 1). Phylogenetic analysis has classified *Arabidopsis* ARFs into four classes, namely Class 1 (six members: ARFA1A/AtARF1, ARFA1B, ARFA1C/AtBEX1, ARFA1D, ARFA1E, and ARFA1F), Class A (two members: ARFB1B and ARFB1C), Class B (one member: ARFB1A), and Class D (two members: ARFD1A and ARFD1B). Class 1 has mammalian relatives and Classes A, B, and D appear only in plants (Singh et al. 2018).

**Table 1:** List of *A. thaliana* gene identifiers of ARF subfamily and cognate GEFs and GAPs.

**ARF GTPase**				**ARF-GEF**			**ARF-AGD**		
ARF	Class I	AT1G23490	ARFA1A/AtARF1	GBF	AT1G13980	GNOM	Class I	At5g61980	AtAGD1
		AT5G14670	ARFA1B		AT5G39500	GNL1		At1g60860	AtAGD2
		AT2G47170	ARFA1C/AtBEX1		AT5G19610	GNL2		At4g13300	AtAGD3
		AT1G70490	ARFA1D	BIG	AT4G38200	BIG1		At1g10870	AtAGD4
		AT3G62290	ARFA1E		AT3G60860	BIG2	Class II	At3g53710	AtAGD6
		AT1G10630	ARFA1F		AT1G01960	BIG3		At2g37550	AtAGD7
	Class A	AT5G17060	ARFB1B		AT4G35380	BIG4	Class III	At4g17890	AtAGD8
		AT3G03120	ARFB1C		AT3G43300	BIG5		At5g46750	AtAGD9
	Class B	AT2G15310	ARFB1A					At2g35210	AtAGD10
	Class D	AT1G02440	ARFD1A				Class IV	At3g07490	AtAGD11
		AT1G02430	ARFD1B					At4g21160	AtAGD12
ARL		AT2G24765	ATARL1					At4g05330	AtAGD13
		AT2G18390	TTN5/ARL2				Class V	At5g54310	AtAGD5
		AT3G22950	ARFC1/AtARL5				Class VI	At1g08680	AtAGD14
		AT3G49870	ARL8A				Class VII	At3g17660	AtAGD15
		AT5G67560	ARL8B						
		AT5G37680	ARL8C						
		AT3G49860	ARL8D						
		AT5G52210	ARLB1/ARFRP1						
SAR		AT1G09180	SAR1						
		AT1G56330	SARA1B						
		AT4G02080	SAR1C						
		AT3G62560	SAR1D						
		AT1G02620	SAR1E						

The table includes the ARF-subfamily members in *Arabidopsis*. The SAR-GEFs and SAR-GAPs are not included and can be found in Brandizzi (2018). ARF-related protein ARFRP1/ARLB1 (AT5G52210) is not included.

*Arabidopsis* Class 1 ARF proteins interact with all *Arabidopsis* ARF-GEFs and are involved in COPI and COPII recruitment in all the major membrane trafficking pathways (Singh et al. 2018). ARF proteins from Classes A and B probably play a non-essential/auxiliary role in support of cargo transport (Singh et al. 2018). Class D ARF proteins participate in membrane trafficking during cell plate formation (Niu et al. 2022). The activating ARF-GEF for Class D proteins remains unidentified; however, evidence of interaction between Class D ARFs and COPI coat proteins were provided (Niu et al. 2022). These results suggest that the major function of ARF proteins, i.e. COP recruitment, is evolutionarily conserved in plants. Also, the site of action reported for mammalian ARFs [cytosol, Golgi, endosomes, plasma membrane (PM), REs] matches with the reported ones for *Arabidopsis* (Golgi, TGN, and PM) (Lee et al. 2002, Takeuchi et al. 2002, Singh et al. 2018, Niu et al. 2022). Some mammalian ARF functions such as activation of phospholipase D, actin rearrangement, or interacting with tethers have not yet been reported for *Arabidopsis* ARFs (Donaldson and Jackson 2011, Sztul et al. 2019, Jackson et al. 2023). The actin rearrangement is particularly interesting as plant cells use actin and myosin perhaps preferentially versus microtubuli and associated motor proteins to move organelles (Brandizzi and Wasteneys 2013). Hence, it is interesting that plants lost ARF6, which is present in the last eukaryotic common ancestor from which Archaeplastida evolved (Vargova et al. 2021). It has been recently reported in a preprint that the ancestral ARF1/ARF6 protein has a more generalized function rather than the specific assigned ones in higher eukaryotes (Preprint: Alves et al. 2025). This contributes to the hypothesis that plant Class I ARFs may have yet undiscovered roles in actin cytoskeleton regulation, and there are reports suggesting ARF-GAPs regulating actin filaments in *Arabidopsis* (Yoo et al. 2008).

A debated role of *Arabidopsis* ARF is in regulating recycling cargo via the putative GNOM-labeled REs. This review details the debate surrounding these GNOM-labeled REs (Qi et al. 2024). ARF-GEF GNOM was initially proposed to be involved in regulating the plant specific GNOM-labeled REs carrying auxin efflux transporter PIN-FORMED 1 (PIN1). Although ARF1 interacts with GNOM, other proteins are probably needed for the formation of these GNOM-labeled REs.

There are some novel roles attributed to *Arabidopsis* ARF proteins such as roles in pollen development, e.g. mediating the transport of important receptor kinases (Zhu et al. 2021). It has been found that ARF proteins can affect the growth rate of *Arabidopsis*, e.g. when a wheat Class I ARF orthologue was overexpressed in *Arabidopsis* (Yao et al. 2009). Further, overexpression has resulted in drought tolerance of *Arabidopsis* by overexpression of a *Spartina alterniflora* ARF orthologue (Karan and Subudhi 2014). Also, *Panicum virgatum* (Pv) PvARFR2 enhanced alkali tolerance in Switchgrass (Li et al. 2024), possibly by interacting with Cytosolic ABA receptor kinase 3 (PvCARK3). This suggests a role of PvARFR2 in regulating membrane trafficking of PvCARK3, though *in vivo* evidence is still lacking. There are reports from the mammalian system that ARFs can interact with enzymes such as phosphatidylinositol kinases to regulate membrane lipid composition (Just and Peranen 2016) and in amino acid sensing (Li and Guan 2023), increasing the repertoire of ARF functions. But there are no such reports yet from plants.

Based on studies from non-plant system, it has been established that SAR1 is recruited by its GEF SEC12, an ER-integral membrane protein lacking the typical Sec7 domain (Singh and Jurgens 2018). SEC12 then activates SAR1 by exchanging GDP to GTP, which leads to the formation of COPII vesicles (Jensen and Schekman 2011, Brandizzi 2018). Crystallographic studies in *Saccharomyces cerevisiae* (Sc) have provided more detailed information on how ScSEC12 can activate ScSAR1 (Joiner and Fromme 2021). The plant COPII system is generally believed to function similarly as in other eukaryotes (Yorimitsu et al. 2014, Brandizzi 2018). Although there is substantial sequence similarity between the five SAR1 isoforms in *Arabidopsis*, unlike the ARF Class I, they are predicted to have both overlapping and unique functions. For example, AtSAR1B and AtSAR1C are involved in different aspects of pollen development (Liang et al. 2020, Liang et al. 2024), while AtSAR1B plays a primary role in female gametophyte development, with AtSAR1A and AtSAR1C playing supporting roles (Liang et al. 2023). AtSAR1E may be a pseudogene as it has missing amino acids corresponding to G1, G2, and G3 motifs (Fig. 2a). For novel roles of SAR proteins related to autophagy, readers are directed to the review by Li et al. (2022).

The section on ARFs and SAR would remain incomplete without discussion on the possible COPI and COPII, TPLATE complex, and clathrin-coated vesicles (CCV) recruited by the subfamily. This has been reviewed earlier in Yorimitsu et al. (2014), Brandizzi (2018), and Singh and Jurgens (2018). Currently, the role of COPII in anterograde transport is under debate (Zouhar et al. 2023). Two mutants of ARF1 (dominant-negative and dominant-active versions) showed that these mutations affect COPI and clathrin localization. Both remained cytosolic in the presence of dominant-negative ARF1, while both were found bound to membranes in large aggregates in the presence of dominant-active ARF1, confirming ARF1’s role in recruiting these proteins along their respective trafficking pathways (Singh et al. 2018). Supporting this data, a proteomic study performed on *Arabidopsis* CCV did not show enrichment of any *Arabidopsis* ARF GTPase or ARF-GEF, except ARF-GAP domain 5 (AtAGD5) (Dahhan et al. 2022), suggesting an ARF-GEF-ARF GTPase (GTP loaded)-ARF-GAP complex is assembled for only a short duration. The ARF-GEF-ARF GTPase complex may rapidly disintegrate and be degraded, whereas the ARF-GAP may remain for a comparatively longer duration in cells. Accordingly, the AtAGD5 was well represented in the proteome (Dahhan et al. 2022).

## ARL Proteins in Plants: Beyond Vesicle Trafficking

Studies in mammalian systems have indicated that ARLs are involved in the maintenance of organelle morphology, MT dynamics, and mitochondrial fusion and cilium biogenesis (Sztul et al. 2019, Vargova et al. 2021). Archaeplastida retained nine ARLs from its last eukaryote common ancestor (Vargova et al. 2021). All ARLs involved in cilia function [ARL3, ARL6, ARL13, and ARL16 (Dewees et al. 2022)] are not present in *Arabidopsis* although they are present in Chloroplastida (Vargova et al. 2021). ARL17 function is still unknown and is also absent in *Arabidopsis*. As cilia were lost during vascular plant evolution (Pazour 2004), this may explain the concurrent loss of associated ARLs.

The remaining four ARL proteins, ARL1, ARL2, ARL5, and ARL8, are sufficient to maintain the *Arabidopsis* ARL cellular function. Studies from mammalian systems suggest that ARL1 and ARL5 are involved in maintaining traffic between the Golgi and endosomes and act as recruiter proteins (Derby et al. 2004, Rosa-Ferreira et al. 2015, Sztul et al. 2019). The endosomes in plants are not clearly defined, and roles of plant endosomes in protein trafficking have been reviewed recently (Qi et al. 2024). Nevertheless, AtARL1 (initially named AtARF3) localizes to Golgi independently of its nucleotide-bound state (Latijnhouwers et al. 2005, Stefano et al. 2006), but requires the myristoylation at Gly-2 (Stefano et al. 2006).

*Arabidopsis* ARL8 has four isoforms. AtARL8 isoforms AtARL8A and AtARL8B (98% amino acid sequence similarity) are closely related paralogs. The presence of two other *Arabidopsis* isoforms AtARL8C (90% amino acid sequence similarity with AtARL8A) and AtARL8D (92% amino acid sequence similarity with AtARL8A) raise a question as to redundant and non-redundant AtARL8 functions. A closer look revealed that AtARL8A (AT3G49870) and AtARL8D (AT3G49860) may have arisen due to a tandem duplication event in chromosome 3. Similarly, genome duplication events may have been involved in giving rise to AtARL8C (AT5G37680) and AtARL8B (AT5G67560). The mammalian ARL8 regulates lysosome traffic and the difference in the mammalian and plant endomembrane system makes it difficult to draw exact parallels on AtARL8 isoform functions (Sztul et al. 2019). Nevertheless, it is an accepted model that, in plants, the prevacuolar compartments, also named multivesicular bodies (MVBs) or late endosomes (LEs) carry the cargoes destined for degradation in the vacuole (Aniento et al. 2022). Proteomic analyses of the tonoplast in barley and *Arabidopsis* identified ARLA1a/AtARL8C, ARLA1c/AtARL8A, and ARLA1d/AtARL8B at the vacuolar membrane (Carter et al. 2004, Endler et al. 2006). Hence, there is a possibility that the *Arabidopsis* ARLs may perform a similar evolutionarily conserved function in regulating the MVB traffic to the tonoplast. It is tempting to speculate that the increased number of AtARL8s helps in increasing the efficiency of cargo transport. But, it is also important to note that both *AtARL8C* and *AtARL8D* expression in *Arabidopsis* is very low compared to *AtARL8A* and *AtARL8B.* Further, AtARL8D may have lost its membrane targeting mechanism due to a shorter N-terminal region without Gly at position 2, and has been considered a pseudogene (Fig. 2a) (Nishikiori et al. 2011). Outside this, ARL8 has been reported as an essential component that aids in viral replication in plants (Nishikiori et al. 2011). The authors hypothesized that plant ARL8 may have an analogous function to HsARF1 during polio infection, by providing a membrane microenvironment where the viral replication complex is kept safe from host defense (Nishikiori et al. 2011).

Mammalian studies indicate that ARL2 is involved in MT biogenesis, mitochondrial fusion, and delivery of cargo to the cilia (Fansa and Wittinghofer 2016, Francis et al. 2016, Sztul et al. 2019). The association of ARL2 with MTs is seen in many species (Francis et al. 2016), and may be considered an evolutionarily conserved function of ARL2. This concept has indirect support in *Arabidopsis*, as *ARL2* mutant embryos have impaired MT formation (Mayer et al. 1999). Experiments performed in different laboratories have identified *Arabidopsis* ARL2 and reported it as TITAN5 (TTN5) or HALLIMASCH (Mayer et al. 1999, McElver et al. 2000). From here on, we use the name TTN5. Homozygous *ttn5* embryos exhibit few, abnormally enlarged cells with enlarged nuclei in both embryo and endosperm, resulting in embryo lethality. The mutant had problems in MT biogenesis (Mayer et al. 1999). Mammalian studies suggest that ARL2 is found in a complex as a 1:1:1 trimer together with tubulin cochaperone cofactor D and β-tubulin in the cytosol (Francis et al. 2017a, Francis et al. 2017b). Interestingly, knockouts of cofactor D mutants (*TITAN1*) have similar embryo-lethal phenotypes in *Arabidopsis* as *ttn5*, but a functional link remains to be proven (Liu and Meinke 1998, Tzafrir et al. 2002).

It was found that similar to HsARL2, TTN5 is present in a GTP-loaded form in cells, presumably not being dependent on an ARF-GEF (Mohr et al. 2024b). TTN5 [wild-type (WT)] and variants (dominant-negative and dominant-active) proteins exhibited slow GTP hydrolysis in an *in vitro* assay, a feature also reported for mammalian ARFs (Turn et al. 2025). This suggested the involvement of a GAP protein in regulating the hydrolysis step of TTN5 GTPase function. A recent mammalian study suggests that in the multi-subunit assembly (involving tubulin co-factors and ARL2 in MT biogenesis), tubulin cofactor C (TBCC) binding catalyzes ARL2-GTP hydrolysis (Taheri et al. 2025). Combining the information of TTN5 involvement in MT biogenesis (at least in dividing cells) with its low inherent GTP hydrolysis activity, one can speculate that it functions in a similar manner as reported for the mammalian system. TTN5 may also be present predominantly as a trimer in the cytosol, perhaps contributing to MT biogenesis with a Tubulin co factor partner (probably TBCC), which could aid in GTP hydrolysis of TTN5. The mammalian studies also suggest that pools of ARL2 are present in different cellular sites (Francis et al. 2016). Paralleling these mammalian data, TTN5 localizes to the PM and various sites of the endomembrane system (TGN and MVB) (Mohr et al. 2024b).

Recently, some light was shed on the probable mechanistic basis of TTN5 function that suggests a role of TTN5 in iron nutrition (Mohr et al. 2024a, Mohr et al. 2024b). Evidence came from biochemical, cell biological, and (probable) cargo analysis. We found evidence of TTN5 binding to IRON TRANSPORTER 1 (IRT1) (Mohr et al. 2024a). Further, the fluorescent puncta of dominant-negative Yellow Fluorescent Protein (YFP)-TTN5 forms in the hypocotyl of *Arabidopsis* had different intracellular mobility compared to YFP-TTN5 WT and a YFP-TTN5 dominant-active form. Accordingly, in *Nicotiana benthamiana,* the colocalization signals between GFP-IRT1 and the mCherry-TTN5 dominant-negative form were significantly higher at the PM (or vesicles associated with/in close proximity to the PM) compared to the same combination of IRT1 and TTN5 WT or dominant-active TTN5 (Mohr et al. 2024a). This suggests that either (i) IRT1 is a “cargo” of TTN5 or (ii) TTN5 regulates “IRT1-containing vesicle” movement. Evidence suggests the first possibility would be a little out of reach in *Arabidopsis* at the moment as ARL3 and phosphodiesterase 6D (partners of mammalian ARL2 for cargo transport) are both absent in *Arabidopsis* (Fansa and Wittinghofer 2016). Regarding the second possibility, the connection can be that MTs mediate cargo trafficking in *Arabidopsis* and TTN5 could act as anchor/or slow down organelles or vesicles in certain constellations (Ambrose et al. 2013, Brandizzi and Wasteneys 2013, Zhong et al. 2024). As a support to TTN5s role in regulating “IRT1-containing vesicle” traffic, it is interesting to note that there is also an interaction between AtSNX1 (SORTING NEXIN 1) and TTN5 (Mohr et al. 2024a). The SNX and MTs have an established role in recycling in *Arabidopsis* (Ivanov et al. 2014, Rodriguez-Furlan et al. 2019, Qi et al. 2024). Similar classes of SNX proteins were also identified as HsARL14 proximity interactors in mammals, suggesting evolutionary conservation of ARL−SNX interactions (Quirion et al. 2024). Additionally, another probable interactor of TTN5 identified was AtPATL2 (PATELLIN2, a SEC14 domain protein) (Mohr et al. 2024a). Higher order *PATL* mutants showed defects in auxin-efflux carrier PIN1 cellular localization (Tejos et al. 2018). All evidences suggest a role of TTN5 (probably via MT biogenesis) acting in tandem with AtSNX (and its interactors) and AtPATLs to mediate the trafficking of “IRT1-containing vesicles” from the TGN/and or PM. Regarding the role of TTN5 in trafficking of “IRT1-containing vesicles” to vacuole there is no clear explanation at this moment (Mohr et al. 2024a). The connection between TTN5−AtSNX1 interaction has also opened up another possibility, mediating TGN-vacuole traffic of IRT1. As indirect evidence, we found fluorescence tagged TTN5 and AtIRT1 constructs colocalizing in MVBs (formed after Wortmannin treatment) (Mohr et al. 2024a). TTN5 does stand at an interesting-juncture in terms of cargo/vesicle sorting with respect to its TGN localization. It probably interacts with other (yet unidentified) proteins that mediate the decision regarding which pathway it would commit to—recycling or vacuolar degradation. Also, TTN5-PATL2-ENHANCED BENDING1 (EHB1) may form a higher order complex with IRT1 (and other PM proteins) and help in vesicle formation. There is very sporadic information on AtARL5 and AtARLB1, and so we do not present them in the review.

## ARF-GEFs in Plants: Recruiting and Mediating GTP Exchange in ARFs

ARF-GEFs mediate the recruitment of the inactive cytosolic GDP-bound ARFs (at least ARF subfamily members ARF- and SAR1-GTPases) to the endomembranes and help in the GDP to GTP exchange (Singh and Jurgens 2018). For mammalian ARLs, the interacting ARF-GEFs are known (Sztul et al. 2019). In *Arabidopsis*, there are still no concrete reports on ARF-GEFs for ARLs. The SAR1-GEFs are different—they remain ER-embedded and have a distinct catalytic domain different from ARF-GEFs (Pasqualato et al. 2002, Brandizzi 2018). *Arabidopsis* ARF-GEFs are grouped in two subfamilies: the Golgi Brefeldin A (BFA)-resistance factor 1 (GBF1) class [members: AtGNOM and GNOM-like (GNL) AtGNL1 and AtGNL2] and BFA-inhibited GEF (BIG) class (members: AtBIG1–5) (Memon 2004, Wright et al. 2014, Brandizzi 2018). A recent preprint report has suggested that GNOM is the ancient ARF-GEF of plants and GNL1 and GNL2 have evolved more recently (Preprint: Singh et al. 2025). In the plant field, it is accepted that AtGNOM and AtGNL1 act primarily in retrograde Golgi to ER trafficking, and AtGNOM is additionally involved in recycling (Richter et al. 2010, Singh and Jurgens 2018). Recent evidence indicates that clathrin-mediated endocytosis and bulk secretion are functional in the *AtGNOM* mutant, suggesting that AtGNOM is not essential for either process (Adamowski et al. 2024). The BIG members also function at the TGN, but regulate secretion and vacuolar traffic (AtBIG1–4) and endocytosis (AtBIG5), besides being active during cytokinesis when the traffic from TGN contributes to form the cell plate (Singh and Jurgens 2018). AtARF1 may be the main target of a majority of ARF-GEFs (Singh et al. 2018). AtGNL2 is considered a functional equivalent to GNOM, performing recycling in pollen (Richter et al. 2011).

All ARF-GEFs share the conserved Sec7 domain, a 200-amino acid catalytic core essential for GDP-GTP exchange (Chardin et al. 1996, Vernoud et al. 2003). Structurally, the Sec7 domain consists of two subdomains forming a superhelix, with a conserved Glu (“glutamic finger”) essential for GEF activity (Casanova 2007, Wright et al. 2014). Mutation of this residue abolished exchange activity of mammalian ARF-GEF protein (Béraud-Dufour et al. 1998, Cherfils et al. 1998). Besides the catalytic Sec7 domain, the GEFs have two domains located in the N-terminus of the Sec7 domain, the dimerization and cyclophilin-binding (DCB) and homology upstream of Sec7 (HUS); and three at the C-terminal side of Sec7, termed homology downstream of Sec7 HDS1, HDS2, and HDS3 (Mouratou et al. 2005, Richter et al. 2010). The BIG members have another additional HDS4 domain (Wright et al. 2014). The functional role of these domains (besides the Sec7) may not be conserved across species. For instance, the N-terminal DCB has been suggested to be involved in ARF-GEF dimerization (by a DCB−DCB interaction) in both *Arabidopsis* (AtGNOM) and human ARF-GEF (HsGBF1 and HsBIG) (Grebe et al. 2000, Ramaen et al. 2007). However, experiments suggest that interaction is also possible between DCB and HUS domains in humans (Ramaen et al. 2007), between DCB and HUS and DCB and SEC7 domains in *Arabidopsis* (Anders et al. 2008). In contrast, dimerization in yeast Sec7 (a yeast specific ARF-GEF) is mediated by C-terminal HSD4 domain (Richardson et al. 2016, Brownfield et al. 2024). Further, even within *Arabidopsis* contrasting reports exists. For example, it appears that AtGNL1 dimerization happens due to DCB−HUS interaction and presumably, there is no DCB−DCB interaction (Brumm et al. 2022). Furthermore, even C-terminal domains may have different functions depending of the ARF-GEF in question, e.g. yeast ARF-GEF Gea C-terminal domain regulates membrane binding (Gustafson and Fromme 2017) and yeast ARF-GEF Sec7 C-terminal HDS2 interacts with the catalytic surface of Sec7 to keep it autoinhibited (Brownfield et al. 2024). Interestingly, AtGNOM C-terminal deletion mutants are mildly developmentally affected compared to the developmentally challenging mutation in the AtGNOM Sec7 domain (Geldner et al. 2004). So clearly, a unified theory for plant ARF-GEF domains cannot be derived from extrapolating information from mammalian systems.

Mammalian studies suggest that upon ARF−GDP interaction with its respective ARF-GEF, the switch regions of the GTPase bind to the hydrophobic groove of the Sec7 domain between the two subdomains (Wright et al. 2014). Then, the glutamic finger inserts into the nucleotide-binding fold (into or in close proximity to the ARF P loop and Mg^2+^-binding site) forced to the nucleotide phosphate by its negative charge (Béraud-Dufour et al. 1998, Mossessova et al. 1998). It leads to destabilization of the coordinating Mg^2+^ and the bound GDP due to steric and electrostatic repulsive effects with the ion and the β-phosphate (Mossessova et al. 2003, Renault et al. 2003, Casanova 2007). Based on affinity studies, the interaction between the β-phosphate and the P loop seems to be of utmost importance for tight nucleotide binding and structural disturbance of the P loop could be the major reason for the decreased affinity (Vetter and Wittinghofer 2001). The interference of the GEF and the concomitant structural change of the P loop are suggested to be the main reason for the reduced nucleotide affinity and its release from the GTPase (Vetter and Wittinghofer 2001). It is also important to note from studies performed in *Arabidopsis* that (i) Sec7 domain alone does not confer specificity to AtARF1 activation (Singh et al. 2018), and (ii) a linker after the helix J (loop>J) in AtGNOM may also play a role in AtARF1 binding (as GNOM loop>J mutant had defects in fully rescuing the *gnom* mutation) (Brumm et al. 2020).

ARF-GEFs exhibit diverse membrane recognition properties (Nawrotek et al. 2023). Recent studies on HsGBF1 suggest that membrane binding induces structural rearrangements, possibly mediated by the HSD1 domain interacting with phosphoinositides, enabling efficient engagement with ARF proteins (Meissner et al. 2023). It is not known if this could be applied for *Arabidopsis* GBF class and BIG class GEFs. It is, however, known that (i) all domains (N-terminal DCB and HUS), Sec7 and C-terminal (HSD1–3) play a redundant role in regulating AtGNOM localization to Golgi and PM (mentioned as AtGNOM-specific PM structures) (Adamowski et al. 2024), (ii) a single amino acid substitution in the HDS2 domain of AtGNOM leads to its PM localization instead of the Golgi (Okumura et al. 2013), and (iii) GNOM dimers mediate the very close proximity insertion of two ARF1 GTPases into membranes that regulate the ARF-mediated vesicle formation (Brumm et al. 2020).

BFA has been extensively used to understand the membrane trafficking events and regulatory mechanism of ARF GTPases and ARF-GEFs (Rodriguez-Furlan et al. 2019). BFA can block the catalytic activity by acting as an uncompetitive inhibitor on the enzyme−substrate complex. It forms a quaternary complex consisting of ARF-GDP-BFA-Sec7 domain, and thereby stabilizes the usually short-lived ARF-GDP-Sec7 intermediate (Jackson and Casanova 2000; experiments performed with human ARF: Mansour et al. 1999; experiments performed in yeast: Peyroche et al. 1999). In *Arabidopsis*, only AtGNL1 and AtBIG3 are BFA-resistant (Anders and Jurgens 2008). Prolonged BFA treatment leads to the formation of “BFA bodies,” which are aggregations of TGNs surrounded by Golgi stacks (Naramoto et al. 2014)

For the history of AtGNOM discovery, readers are directed to Richter et al. (2010). Some partial loss of function mutations of AtGNOM helped in understanding its role in AtPIN1 recycling (Geldner et al. 2004, Okumura et al. 2013). For other PIN proteins, AtGNOM may not be the sole responsible ARF-GEF (Pukhovaya et al. 2024). AtGNOM is also responsible for the PM localization of D6 PROTEIN KINASE, strengthening AtGNOMs role in auxin-mediated development (Barbosa et al. 2014). Other established targets/cargo of GNOM are Polarity of REQUIRES HIGH BORON 1 (AtBOR1), receptor kinases INFLORESCENCE AND ROOT APICES RECEPTOR KINASE (IRK) (along with multiple ARF-GEFs), and BREAKING OF ASYMMETRY IN THE STOMATAL LINEAGE (AtBASL) (Yoshinari et al. 2021, Wang et al. 2022, Rodriguez-Furlan et al. 2024). A recent reported target/cargo exclusive for AtGNL1 is KINASE ON THE INSIDE (the partner of AtIRK to repress root cell proliferation) (Rodriguez-Furlan et al. 2024).

Two pathways for cargo recycling from the TGN have been described in plants (i) by AtGNOM-labeled REs and (ii) MT-SNX mediated vesicles (Qi et al. 2024). However, it has to be noted that secretion and TGN recycling pathways might converge, latest at the Golgi-independent TGN (Shimizu et al. 2021, Qi et al. 2024), and that both SNX and GNOM pathways recycle PINs. We therefore propose that SNX and GNOM might be sequential steps in the same recycling pathway at the TGN. Indeed, no evidence of direct physical interaction has been reported between AtGNOM and SNXs, however, recent reports have revealed additional partner proteins for both AtGNOM (PRENYLATED RAB ACCEPTOR-1 DOMAIN FAMILY and Flippase Aminophospholipid ATPase3) and AtSNX1 (TTN5) (Zhang et al. 2020, Wang et al. 2022, Mohr et al. 2024a). Further analyses of these and additional interactors will support the efforts to better understand protein recycling at the TGN.

There have been very little new mechanistic insights on the BIG group of ARF-GEFs since an earlier summarizing report (Singh and Jurgens 2018). BIGs are now also considered to be involved in PIN trafficking (Zhang et al. 2020), although the evidence provided in this report suggest that AtBIG3 and AtBIG5 function redundantly in endocytosis (Suo et al. 2021).

## ARF-GAPs in Plants: Performing GTP Hydrolysis

ARF-GAPs help in the GTP-hydrolysis and complete the ARF GTPase cycle. There are three previous phylogenetic classification systems for *Arabidopsis* ARF-GAPs (Vernoud et al. 2003, Min et al. 2007, Singh and Jurgens 2018). In this report, we refer to (Singh and Jurgens 2018) for nomenclature. Accordingly, the 15 *Arabidopsis* ARF-GAPs are grouped in seven classes (Classes I−VII). Mammals in comparison have more reported ARF-GAPs, but these could be due to the inclusion of non-canonical ARF-GAPs in the list (Sztul et al. 2019, Nikolatou et al. 2023). The AGD is ~70 amino acids long, features a conserved zinc finger motif (CXXCX_16–17_CXXC) near the N-terminus, essential for GAP activity. Mutational studies have also identified a conserved Arg residue critical for catalyzing GTP hydrolysis (Vitale et al. 1998, Randazzo et al. 2000, Memon 2004).

*Arabidopsis* Class I ARF-GAPs (AGD1–4) are similar in sequence to mammalian ACAPs ARF-GAPs, which are functional in a wide range of subcellular compartments (Singh and Jurgens 2018, Sztul et al. 2019). Similarly, *Arabidopsis* Class I ARF-GAPs also have different localization patterns reported (Singh and Jurgens 2018). A major function attributed to AGD3/VAN3/SCARFACE is in endocytosis (together with GNOM) (Singh and Jurgens 2018). However, it is important to note that a quadruple mutant of Class I ARF-GAP is required to phenocopy a mutant with reduced AtGNOM activity, suggesting that *in planta* the loss of *AGD3* could to some extent be compensated by the other group members (Naramoto et al. 2010). Separately, AtBOR1 endocytosis is regulated by AGD3 along with other proteins that are not ARF-GAPs (Pukhovaya et al. 2024). A novel role in autophagy has been indicated for AtAGD1 but the mechanistic details are still lacking (Cheng et al. 2021).

The Class II (AGD6–7) and two of the Class III (AGD8–9) probably have a role in regulating the anterograde transport via regulating ARF1 (Singh and Jurgens 2018). It is important to note here *agd8agd9* double knockout plants are embryo-lethal, and it can be inferred that both play important role in basic cellular functions (Min et al. 2013). The Class III AtAGD10 is localized to the Golgi and may have a functionally different role than AGD8 and AGD9, specific to pollen and root hairs (Song et al. 2006, Boavida et al. 2009). Class II (ADG7) and Class III (ADG8–9) are also involved in maintaining Golgi function (Min et al. 2007, Min et al. 2013).

The Class IV AGDs (AGD11–13) are considered to be plant-specific and can target peroxisomes (AGD11/CML3) and TGN (AGD12/aka ARF-GAP ZAC). An *AtAGD13* mutation causes gametophytic lethality (Renak et al. 2012). It is also important to note that this class has the C2 domain that it shares with C2-DOMAIN ABA-RELATED (CAR) proteins (that lack the ARF-GAP domain) (Cui et al. 2023). There may be a connection between CAR protein EHB1 and AtAGD12 (Dummer et al. 2016). In separate reports, AtEHB1 has been shown to be involved in AtIRT1 trafficking along with TTN5 (Khan et al. 2019, Mohr et al. 2024a). Recently, two studies from rice [*Oryzae sativa* (Os)] focused on OsAGD12 (Han et al. 2024, Zhao et al. 2024). Although the nomenclature makes it very difficult, the sequence similarity with AGD12 indicates that OsAGAP (59.4%) and OsZAC (60%) are the same OsAGD12 orthologue (Han et al. 2024, Zhao et al. 2024). Studies suggest opposing roles for OsAGD12 in regulating root development and grain number. OsAGD12 may interact with rice proteins quinone reductase RoLe1 during root development, and a flowering locus T-like 1 protein for regulating grain size.

Class V (sole member AGD5/NEV) is also plant-specific. The protein is located at the TGN and may be involved in regulating vacuolar traffic (Singh and Jurgens 2018). No new information has been reported for Class VI (AGD14) and Class VII (AGD15) (Singh and Jurgens 2018). The *Arabidopsis* SAR-GAPs (more popularly known as SEC23) have been extensively reviewed in Brandizzi (2018).

## Perspectives: Increasing the Scope of ARF GTPase Network in Plants

The major focus of the ARF GTPase network in plants has always been addressed from the perspective of understanding probable roles in cargo sorting. Despite the significant progress made in the field, they are still rather poorly described. Plant biologists often infer functional and structural features from yeast and mammalian homologs. This has often proven to be a successful approach. However, given the examples described in this review regarding the mechanistic differences existing in plant cells, there may be significant pitfalls in using this approach. For most ARF family members, the specific cellular functions, localizations, and effectors remain unknown or not proven, despite existing knowledge from other systems. Critical is also understanding the non-trafficking roles within the family. While traditionally linked to vesicle transport, available evidence suggests that many of these proteins are involved with cytoskeleton regulation, lipid transport, and organelle positioning, though these pathways are not yet well understood in plants.

In the context of TTN5 at this point, we have several described and suspected functions ranging from basic molecular properties to physiological effects. Bridging this enormous gap would mean understanding more of the molecular role that TTN5 plays in the organization of the cell. Could it be that it acts as a scaffold that recruits proteins involved in compartment biogenesis and function as suggested by its presence at multiple endomembrane compartments and their changed behavior depending on TTN5 activation state? If yes, does it also perform this as part of regulating specific proteins, e.g. recruiting effectors to modulate transporter function at the PM, as suggested for IRT1? Being linked to endomembrane compartment dynamics and at the same time to MT biogenesis, could it be involved in potential cross-talk in cytoskeleton organization? We also speculate that there is a link between GNOM and TTN5 (Fig. 3). It would be interesting to investigate if it can act in parallel to GNOM or potentially be part of the same process and maybe even the same protein complex. As a step in addressing these and further open questions, a crucial point would be to collect an inventory of the TTN5 protein interacting partners. This would present us with a database of potential processes, signals and effectors converging at TTN5 and will allow formulating specific goals.

**Figure 3:**
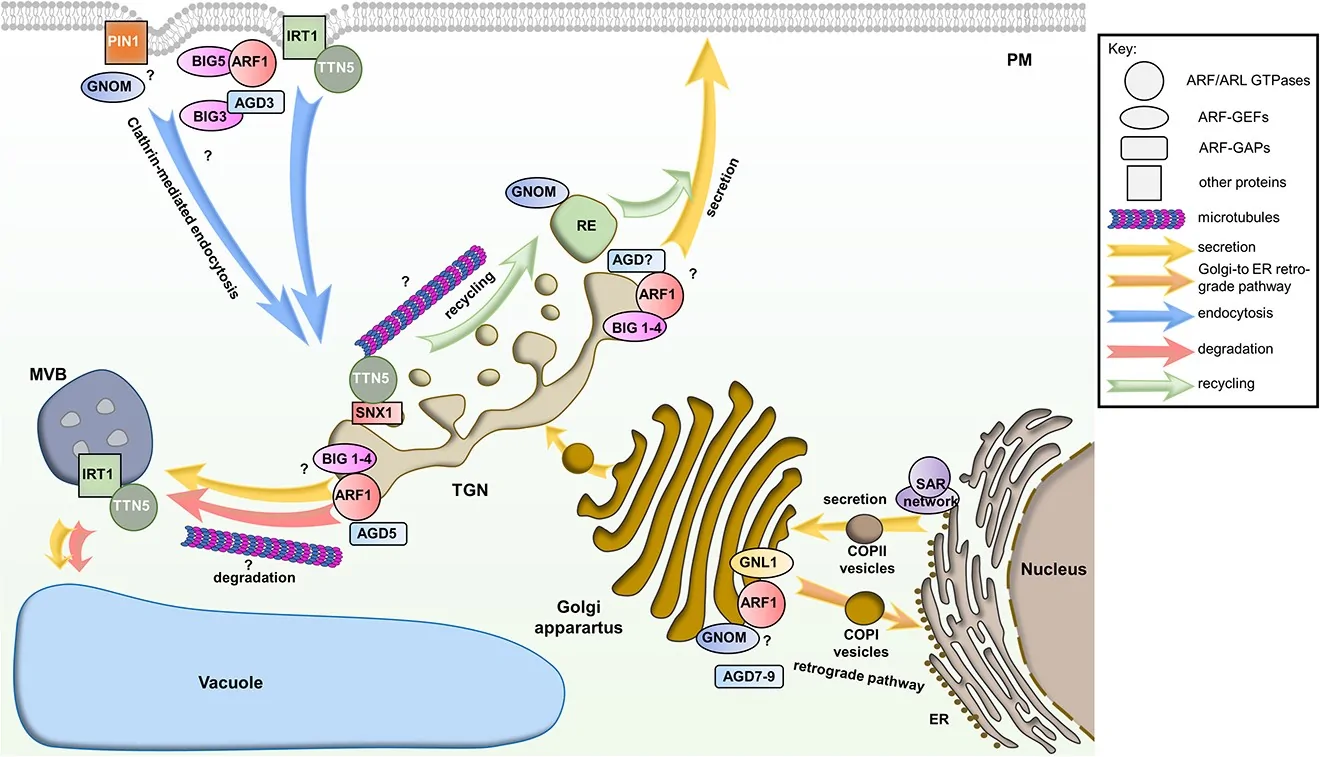
ARF subfamily in plant endomembrane trafficking. The panel presents a short summary and outlines unclarities in the role of ARF proteins and their regulators in the intracellular trafficking of plant cells, mainly based on the *Arabidopsis* data. Processes such as autophagy in response to nutrient deprivation/stress, and cargo delivery during cytokinesis where ARF proteins are known to be involved, or multiprotein complexes of non-ARF-related proteins, such as SNX1, are not depicted here. Uncertainties over the involvement of proteins in different processes are noted with question marks. The ARF and ARL GTPases are represented by circles, ARF-GEFs by ellipses, ARF-GAPS by rounded corners rectangles, and all other proteins by rectangles. The yellow arrows represent secretion and anterograde pathway (collectively labeled as ‘secretion’), orange arrows represent retrograde pathway, blue arrows represent endocytosis, green arrows represent recycling, and red arrows represent degradation pathway. The network of SAR-GEFs-SAR-GTPases (SAR network) regulates anterograde traffic (secretion pathway, yellow arrow) between the endoplasmic reticulum and Golgi apparatus. Class 1 ARFs, together with GNOM and GNL1 ARF-GEFs, regulate the retrograde traffic towards ER (retrograde pathway, orange arrow). AGD7/AGD8/AGD9 may be the ARF-GAPs involved in regulating ARF1 activity in this pathway. BIG1 to BIG4 ARF-GEFs mediate post Golgi secretion and vacuolar traffic (degradation pathway, red arrows) at the TGN. The ARF-GAP AGD5 is involved in regulating the trafficking towards the vacuole. ARF1 may be the GTPase involved in both processes and the involvement of ARF-GEFs and AGDs is assumed. GNOM was suggested to play a role in endocytosis (blue arrow) of PM-localized PIN auxin transporters via clathirin-mediated endocytosis. ARF1, BIG5, and potentially BIG3 may also regulate endocytosis. AGD3 is also involved in endocytosis, but it is important to note that all four Class I ARF-GAPs need to be knocked out to observe the developmental defects associated with GNOM function. GNL1 and other known cargo of the network are not indicated. A direct connection between ARF-GTPases, ARF-GEFs, and ARF-GAPs for endocytosis/recycling (green arrow) is unknown, and is assumed. At the TGN, endocytosed material can either be recycled (green arrows) or proceed to vacuolar degradation (red arrows). The role of the ARF network in the recycling process is not yet clear; however, it involves the GNOM-labeled RE. Some of the already described proteins, such as ARF1, BIGs, and AGD5, might also take part in the sorting process at the TGN. The sorting nexin SNX1 is known to form complexes facilitating either degradation or recycling at the TGN. The ARL TTN5 interacts with proteins, such as SNX1 and the IRT1. TTN5 may play a role in MT organization and has been suggested to link cargo complexes, sorting machinery, and the cytoskeleton at the PM and the TGN. It was also shown to colocalize and could potentially interact with IRT1 in LE compartments and PM.

The current paradigm of ARF-based vesicle trafficking suggests an ARF-GEF-mediated recruitment of ARF GTPases to the site of vesicle formation and ARF-GAP-mediated completion of the GTPase cycle to complete the cargo (and COPI and COPII)-bound vesicle release. It has been argued that ARF-GAPs mediate cargo sorting, and not terminate ARF function (Turn et al. 2025). In this context, the recent findings on GAP stability via protein interactions support that idea in plants (Han et al. 2024, Zhao et al. 2024). Although biochemical data on most plant ARF/ARL/SAR GTPases are lacking, the data from TTN5 suggest plants and mammalian ARF GTPases have inherently slow GTP-hydrolyzing activity (Mohr et al. 2024a, Turn et al. 2025). If this is the case for all ARF proteins, then GAPs play a much larger role in ARF GTPase physiology. It is equally important to note that novel functions of ARF subfamily members are being reported, e.g. in pectin distribution (Wachsman et al. 2020) or plasmodesmata regulation (both by GNOM) (Linh and Scarpella 2022), suggesting the versatile nature of the network.

Finally, the questions in Box 1 are critical to get more detailed answers to the functional role of the ARF family in plants.

Box 1 Outstanding/Open questions.

1.  Are other cargoes of GNOM endocytosed via the ARF network-based vesicles or are they different, as recently suggested for PIN1?
2.  Is there a chance that crosstalk between the GNOM and MT network mediates recycling?
3.  How versatile is TTN5 in terms of its regulatory role in plant cells?
4.  Are ARF-GAPs a critical switch that provides temporal regulation and/or pathway specificity to the ARF network?

## Data Availability

No new datasets were generated or analyzed in this study.
